# COVID-19 and Neutrophils: The Relationship between Hyperinflammation and Neutrophil Extracellular Traps

**DOI:** 10.1155/2020/8829674

**Published:** 2020-12-02

**Authors:** Leandro Borges, Tania Cristina Pithon-Curi, Rui Curi, Elaine Hatanaka

**Affiliations:** ^1^Instituto de Ciências da Atividade Física e Esportes (ICAFE), Universidade Cruzeiro do Sul, São Paulo, SP, Brazil; ^2^Instituto Butantan, São Paulo, SP, Brazil

## Abstract

Coronavirus disease 2019 (COVID-19) is a virus-induced respiratory disease that may progress to acute respiratory distress syndrome (ARDS) and is triggered by immunopathological mechanisms that cause excessive inflammation and leukocyte dysfunction. Neutrophils play a critical function in the clearance of bacteria with specific mechanisms to combat viruses. The aim of this review is to highlight the current advances in the pathways of neutrophilic inflammation against viral infection over the past ten years, focusing on the production of neutrophil extracellular traps (NETs) and its impact on severe lung diseases, such as COVID-19. We focused on studies regarding hyperinflammation, cytokine storms, neutrophil function, and viral infections. We discuss how the neutrophil's role could influence COVID-19 symptoms in the interaction between hyperinflammation (overproduction of NETs and cytokines) and the clearance function of neutrophils to eliminate the viral infection. We also propose a more in-depth investigation into the neutrophil response mechanism targeting NETosis in the different phases of COVID-19.

## 1. Introduction

The World Health Organization (WHO) established the coronavirus disease 2019 (COVID-19) as a pandemic on March 11, 2020. Severe acute respiratory syndrome coronavirus 2 (SARS-CoV-2) is a member of the coronavirus family, a class of enveloped viruses with a positive-sense single-stranded RNA genome. This virus can cross species barriers and induce illnesses ranging from the usual cold to severe interstitial pneumonia, respiratory failure, and septic shock [1]. While there is a global effort in the development of vaccines and improvement of diagnostic methods [2, 3] and therapies that relieve the symptoms and prognosis of COVID-19 patients under severe infection [4], there remain gaps in our understanding of the pathophysiology of COVID-19 related to innate immunity.

In a scenario where patients with severe COVID-19 could develop dysfunction of the immune response that aggravates the hyperinflammation [5, 6], it is hypothesized that neutrophils can amplify pathological damage or control other cell subsets depending on the infection features. Therefore, to use the potential of NETs with minimal damage to the hosts, there must be a right balance of NET formation and reduction of the amount of NETs that accumulate in tissues [7].

Notwithstanding the rapid progress in the field, there are many critical unknown features of neutrophils in fighting viral infections. We highlighted the current progress in the pathways of neutrophilic inflammation in viral infection, with a focus on the release of NETs and its influence on lung disease. The knowledge summarized in this study should benefit researchers in integrating neutrophil biology to design new and more efficient virus-targeted interventions concerning COVID-19.

## 2. Hyperinflammation

Although a well-regulated innate immune process is the first protection action against viral infections [8], in severe COVID-19 condition occurs hyperinflammation (“cytokine storm”) that might lead to the acute respiratory distress syndrome (ARDS) [6, 9].

Cytokines play a relevant function in immunopathology during virus infections. The host-viral interactions are established via host identification of pathogen-associated molecular patterns (PAMPs) of the virus [10]. This identification occurs through host pattern recognition receptors (PRRs) manifested on innate immune cells (e.g., neutrophils, dendritic cells, epithelial cells, and macrophages) [11], and the recognition of PAMPs and viral danger-associated molecular patterns (DAMPs) by conserved PRRs marks the first line of defense against pathogens, involving toll-like receptors (TLRs) [11].

TLR stimulation activates the nuclear factor-*κ*B (NF-*κ*B) signaling cascade, causing the production of inflammatory markers from monocytes (interleukin- (IL-) 1, tumor necrosis factor-alpha (TNF-*α*), and IL-6) to control virus infections [8] by direct antiviral pathways and the recruitment of other leukocytes [10]. Moreover, the exacerbated oxidative stress induced by elevated concentrations of cytokines, along with reduced concentrations of interferon *α* and interferon *β* (IFN-*α*, IFN-*β*), influences the severity of COVID-19 [12].

Several mediators control the release of chemoattractants and neutrophil activity [10], and studies have demonstrated that higher values of proinflammatory markers are related to extensive lung damage and pulmonary inflammation in MERS-CoV [13] and ARDS infection [14]. COVID-19 in the severe state exhibits a cytokine storm with elevated plasma levels of chemokine ligand 2 (CCL2), IFN*γ*, IFN*γ*-inducible protein 10, G-CSF, chemokine C-C motif ligand 3 (CCL3), IL-1*β*, IL-2, IL-6, IL-7, IL-8, IL-10, IL-17, and TNF-*α* [12, 15]. Nucleotide-binding oligomerization domain- (NOD-) like receptor and increased plasma levels of chemokines and cytokines in COVID-19 patients relate to the severity of the disease rather than did those nonsevere patients [5]. In this sense, Huang et al. [15] found that patients in the intensive care unit (ICU) with laboratory-confirmed COVID-19 infection had higher plasma levels of IL-2, IL-7, IL-10, interferon-inducible protein 10, granulocyte colony-stimulating factor, CCL2, CCL3, and TNF-*α* when compared with non-ICU patients [15].

## 3. Neutrophils: The First Cell Recruitment

Neutrophils are innate immune cells with a brief lifespan after leaving the bone marrow and exist in a quiescent, primed, or active state. These leukocytes are the leading players in innate immunity since they are among the first innate leukocytes recruited during infections [16]. The primary function of neutrophil is clearance of pathogens and debris through phagocytosis [17]. They also have a distinct array of other immune roles, such as the liberation of NETs for viral infection inactivation [18] and cytokine production to restrict virus replication [16].

The release of neutrophil-chemoattractive elements and the resulting recruitment of neutrophils are a global host response to viral infection [19]. In this scenario, the neutrophil cell membrane also expresses a complex array of receptors and adhesion molecules for various ligands, including immunoglobulins, membrane molecules on other cells, and cytokines [20].

In addition to the trafficking to infection places to phagocytize viruses, the neutrophils can initiate, enlarge, and/or repress adaptive immune effector processes by promoting bidirectional cross-talk with T cells [21, 22]. Following the acute inflammation arising from immunological processes, such as viral infections, neutrophils with decreased expression of CD62L weaken T cell migration via the CXCL11 chemokine gradient by releasing H_2_O_2_ into an immunological synapse [23]. Thus, neutrophils that uncovered viral antigens can home to draining lymph nodes, acting as antigen-presenting cells (APC) [24]. Hufford et al. [25] evidenced that neutrophils expressing viral antigen as an outcome of direct infection by influenza A virus (IAV) display the most potent APC activity and that viral antigen-presenting neutrophils infiltrating the IAV-infected lungs act as APC for effector CD8(+) T lymphocytes in the infected lungs [25]. Neutrophils recruit the T cell molecular mechanism during the influenza virus infection and associate to CXCL12 reservoirs left behind. CD8+ T cells follow the chemoattractant trail left behind by neutrophil uropods to the influenza virus infection site [26].

Decreased cell number or impaired leukocyte function can play a part in advance of mild to severe clinical disease conditions [16]. Regarding the new coronavirus, the neutrophil-to-lymphocyte ratio (NLR), a well-known marker of infection and systemic inflammation, has evidenced an enhanced inflammatory response in COVID-19 patients [5]. Since the ARDS is the primary cause of mortality in patients with COVID-19, the elevated NLR values suggest a poor prognosis in COVID-19 disease [27], especially severe COVID-19 compared to mild patients. Sun et al. [28] studied 116 patients with COVID-19 and showed a higher NLR [28]. The authors compared severe COVID-19 patients admitted to the ICU with others or severe patients not admitted to the ICU. They reported that COVID-19 patients have the lowest count of lymphocytes and the highest neutrophil count and NLR [28]. Wang et al. [29] also showed that several COVID-19 patients have a rising neutrophil count and a falling lymphocyte count during the severe phase [29]. Similarly, Barnes et al. [30] found extensive neutrophil infiltration in pulmonary capillaries from a COVID-19 patient [30]. Nevertheless, even though severe cases of COVID-19 appear to be related to increased NLR levels [5], whether NLR could be an independent predictor of mortality in COVID-19 patients still requires investigation.

## 4. Neutrophil Extracellular Traps (NETs) and Viral Infection

Neutrophils can develop a sophisticated network of DNA called NETs through NETosis, a liberation of web-like structures of nucleic acids wrapped with histones that detain viral particles [31]. Upon discovery, the researchers believed that the production of NETs defended only against fungi and bacteria [32]. However, the NETosis process plays an important function in the response to viral diseases [33], thereby protecting the host during the virus response by trapping and eliminating distinct pathogens [31].

The formation of NETs is a controlled process, even though the related signals remain unknown. NETosis is conditional on the production of reactive oxygen species (ROS) by nicotinamide adenine dinucleotide phosphate oxidase (NADPH oxidase) [34]. There is evidence of NETosis produced in a ROS-independent mechanism [35]. In general, the NETosis process includes the release of nuclear chromatin lined with effector proteins and peptidyl arginine deiminase type IV (PAD4) activation [36]. After stimulation, the neutrophil nuclear envelope disintegrates to enable the mixing of chromatin with granular proteins [37]. Myeloperoxidase (MPO) and neutrophil elastase (NE) stimulate chromatin condensation and deteriorate histones [38]. In the presence of histone hypercitrullination, PAD4 mediates chromatin decondensation, and the DNA-protein complexes are released extracellularly as NETs [37]. Therefore, differently from apoptosis or necrosis, both the granular membrane and nuclear membrane deteriorate during NETosis, whereas plasma membrane integrity remains [36].

The overproduction of NETs induces lung tissue damage by NETosis-related enzymes such as NE and MPO [39]. Uncontrolled NET production correlates with disease gravity and lung injury extension. For instance, NETosis markers are related to bacterial burden and local inflammation in the lung [40] and patients with pneumonia-associated ARDS have neutrophils in a “primed” condition to generate NETs [41].

During chronic obstructive pulmonary disease aggravation, the production of NETs increases in people with acute respiratory failure [39] and in ARDS patients [40, 42]. The elevated NET production, as noted in patients with severe IAV infection [43], increased injury to the pulmonary endothelial and epithelial cells [44], directing to severe pneumonia. Zhu et al. [43] also noted that the production of NETs positively correlates with multiple organ dysfunction syndromes [43].

The inflammatory process is a trigger for thrombotic complications usually noted in COVID-19 patients, and the immunothrombotic dysregulation seems to be an important key marker for the disease severity [45]. Skendros et al. [46] found that complement activation potentiates the platelet/NET/tissue factor/thrombin axis during SARS-CoV-2 infection [46]. In contrast, Nicolai et al. [47] noted that, in COVID-19, inflammatory microvascular thrombi are found in the kidney, lung, and heart, containing NETs related to the fibrin and platelets. In blood, Nicolai et al. also show that COVID-19 patients have neutrophil-platelet aggregates and a different platelet and neutrophil activation pattern, which alters with the disease severity [47]. Middleton et al. [48] also found that plasma MPO-DNA complexes increased in COVID-19 and that the elevated NET formation correlates with COVID-19-related ARDS. Together, these findings suggest the timely application of therapeutic strategies that can disrupt the vicious cycle of COVID-19 immunothrombosis/thromboinflammation by targeting neutrophil activation and NET formation.

In addition to the physical containment promoted by NETosis [33], NETs contain DNA, modified extracellular histones, proteases, and cytotoxic enzymes that allow neutrophils to centralize lethal proteins at infection sites [7]. The mechanisms of NETs' release in the viral response seem to involve neutrophil NE production attributed to the change of macrophage role by the cleavage of TLRs [49]. A range of stimuli, including toxic factors, viruses, and proinflammatory cytokines, such as TNF-*α* and IL-8, can lead neutrophils to release NETs [7, 33]. Mechanisms that determine strain specificity to induce NETosis formation during viral infection are still unknown.

Lung inflammation is the leading cause of the life-threatening respiratory complication at the severe levels of COVID-19 [50]. Veras et al. [51] investigated the potentially detrimental function of NETs in the pathophysiology of 32 hospitalized severe COVID-19 patients and found that the levels of NETs increase in tracheal aspirate and plasma from patients with COVID-19 and their neutrophils naturally produced more significant concentrations of NETs [51]. The authors also reported NETs in the lung tissue specimens from autopsies of COVID-19 patients. *In vitro*, they noted that viable SARS-CoV-2 cause NET production by healthy neutrophils through a PAD-4-dependent manner and that NETs produced by SARS-CoV-2-activated neutrophils instigated lung epithelial cell death [51]. Zuo et al. [52] also investigated sera from COVID-19 patients and found higher cell-free DNA, myeloperoxidase-DNA (MPO-DNA), and citrullinated histone H3 (Cit-H3) [52]. *In vitro*, they also noted that sera from COVID-19 patients trigger NET release from control neutrophils [52].

**Figure 1:**
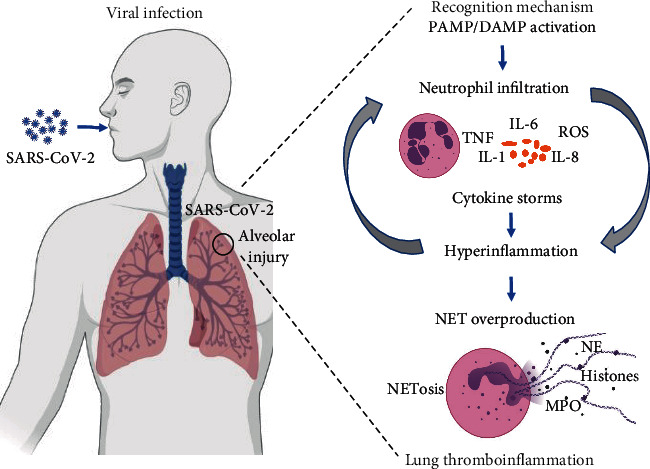
The interaction hypothesis between neutrophil and hyperinflammation in COVID-19. After the host-viral interaction, the virus signaling leads to a cascade of interactions between the virus recognition mechanism, neutrophil activation, and inflammatory stimuli. The NETosis process can protect the host during the virus response or exacerbate lung hyperinflammation in COVID-19 patients. The figure is made with BioRender (https://app.biorender.com/). Abbreviations: SARS-CoV-2: severe acute respiratory syndrome coronavirus 2; PAMP: pathogen-associated molecular pattern; DAMP: danger-associated molecular pattern; TNF: tumor necrosis factor; IL-6: interleukin-6; IL-1: interleukin-1; IL-8: interleukin-8; ROS: reactive oxygen species; NE: neutrophil elastase; MPO: myeloperoxidase.

Although the literature does not report direct evidence linking NETs and SARS-CoV2 clearance, virus entrapping by NETs was already found in syncytial respiratory virus infection [53] or influenza [54]. Furthermore, in virus infection, NETs are efficient to block viruses at the infection site, entrapping them in a DNA web [22]. Therefore, the NETosis process induced by the virus could operate as a double-edged sword: on the one hand, there are essential and efficient mechanisms for trapping the virus [55], and on the other, there are highly intense immunological and inflammatory processes triggered by NET release causing damage to the organism [7]. These interactions could influence the COVID-19 symptoms in the relationship between hyperinflammation (overproduction of NETs and cytokine storm) and the function of neutrophils to destroy the viral infection (Figure 1).

## 5. Concluding Remarks and Future Directions

The exacerbated NET formation can drive to a cascade of inflammatory reactions that destroys surrounding tissues, favors microthrombosis, contributes to the progress of cancer cell metastasis, and results in permanent damage to the pulmonary, cardiovascular, and renal systems [56]. Whether by coincidence or a cause-and-effect relationship, these organs are affected in the severe state of the COVID-19 disease [57, 58]. The uncontrolled and poorly acknowledged host response regarding the cytokine storm is one of the major causes of severe COVID-19 conditions [12]. In this pandemic scenario, there is a compelling need to investigate the mechanisms associated with hyperinflammation process and NET production in response to COVID-19.

The NLR is an independent risk factor for severe COVID-19 [27], and neutrophilia forecasts poor outcomes in COVID-19 patients [29]. In this sense, new frontiers in NET assessment regarding COVID-19 may be expressed by analyzing NETosis directly after sputum induction or after bronchoscopy using the bronchial alveolar fluid of COVID-19 patients [42]. Since patient samples usually become accessible at the hospital, it could investigate whether the existence of NETs is associated with the severity of COVID-19.

Treatments using NET-targeting approaches, although would not directly target the new coronavirus, could reduce the damage caused by hyperinflammation [59], thereby decreasing the disease's severity and avoiding invasive mechanical ventilation, consequently diminishing mortality. Drugs that target NETs include inhibitors of the molecules necessary for NET formation, such as gasdermin D [60], PAD4 [61], and NE [62]. Studies on treatment of inflammatory state in COVID-19 patients with NET inhibitors are still in development (please see Table 1).

Caution is needed to define which people would advantage from suppressing the neutrophil response and which would help more from a strengthened neutrophil action during viral infections. Despite prior studies linking pulmonary diseases to aberrant NET formation ^[^3, 4^]^, our understanding of NETosis mechanisms in viral infection is still limited.

The hyperinflammation is related to the severity of COVID-19 by influencing the pulmonary inflammation [12]. Neutrophils exhibit an intense response to virus infection, promoting bidirectional cross-talk with T cells [21]. Neutrophils also express a complex array of receptors and adhesion molecules for various ligands, including immunoglobulins and inflammatory markers [20]. In this sense, severe cases of COVID-19 appear to be related to increased NLR levels [5], and treatments using NET-targeting approaches have the potential to decrease the damage caused by hyperinflammation [40, 41]. The researchers should consider hyperinflammation in the different phases of COVID-19, neutrophil response mechanisms, and NETosis.

## Figures and Tables

**Table 1:** Interventional studies registered at the ClinicalTrials.gov database relating the treatment of COVID-19 with NET inhibitors.

NCT identifier	Status	Location	Study type	Condition or disease	Intervention and phase	Primary outcome	Estimated completion date
NCT04409925	Not yet recruiting	Canada	Nonrandomized pilot study	COVID-19	Dornase Alfa Phase: 1	(1) Rate of all adverse events	January 2021
NCT04359654	Not yet recruiting	United Kingdom	Randomized clinical trial	COVID-19 Hypoxia	Drug: Dornase Alfa Phase: 2	(1) Change in inflammation (C-reactive protein)	November 2020
NCT04445285	Recruiting	United States	Randomized clinical trial	COVID-19	Dornase Alfa Phase: 2	(1) All-cause mortality (2) Systemic therapeutic response	February 2021
NCT04432987	Recruiting	Turkey	Randomized clinical trial	COVID-19	Dornase Alfa Phase: 2	(1) Clinical improvement and inflammatory markers in blood (2) Intubation or extubation	September 2020
NCT04402944	Not yet recruiting	United States	Randomized clinical trial	COVID-19	Dornase Alfa Phase: 2	(1) Ventilator-free days	December 2021
NCT04322565	Recruiting	Italy	Randomized clinical trial	COVID-19 Pneumonia	Colchicine Phase: 2	(1) Clinical improvement (2) Hospital discharge	December 2020
NCT04326790	Recruiting	Greece	Randomized clinical trial	COVID-19	Colchicine Phase: 2	(1) Time to clinical deterioration (2) Concentration of cardiac troponin	September 2020
NCT04402970	Recruiting	United States	Nonrandomized clinical trial	COVID-19 ARDS	Dornase Alfa Phase: 3	(1) Improvement in partial pressure of O_2_ to fraction of inspired O_2_ ratio	May 2022
NCT04355364	Recruiting	France	Randomized clinical trial	COVID-19 ARDS	Dornase Alfa Phase: 3	(1) Occurrence of at least one grade improvement (ARDS scale severity)	August 2020
NCT04322682	Recruiting	United States	Randomized clinical trial	COVID-19	Colchicine Phase: 3	(1) Number of participants who die or require hospitalization	December 2020
NCT04328480	Recruiting	Argentina	Randomized clinical trial	COVID-19	Colchicine Phase: 3	(1) Number of participants who die (all-cause mortality)	August 2020

Retrieved October 30, 2020, from https://www.https://www.clinicaltrials.gov/ct2/home. Abbreviations: NCT: National Clinical Trial; O_2_: oxygen; ARDS: acute respiratory distress syndrome.

## Data Availability

The data supporting this narrative review are from previously reported studies and datasets, which have been cited.
